# Exposure to Phthalates in Neonatal Intensive Care Unit Infants: Urinary Concentrations of Monoesters and Oxidative Metabolites

**DOI:** 10.1289/ehp.8926

**Published:** 2006-04-26

**Authors:** Jennifer Weuve, Brisa N. Sánchez, Antonia M. Calafat, Ted Schettler, Ronald A. Green, Howard Hu, Russ Hauser

**Affiliations:** 1 Department of Environmental Health and; 2 Department of Biostatistics, Harvard School of Public Health, Boston, Massachusetts, USA; 3 Centers for Disease Control and Prevention, Atlanta, Georgia, USA; 4 Science and Environmental Health Network, Boston, Massachusetts, USA; 5 Channing Laboratory, Department of Medicine, Brigham and Women’s Hospital, Harvard Medical School, Boston, Massachusetts, USA

**Keywords:** di(2-ethylhexyl) phthalate (DEHP), infants, mono(2-ethylhexyl) phthalate (MEHP), mono(2-ethyl-5-hydroxylhexyl) phthalate (MEHHP), mono(2-ethyl-5-oxohexyl) phthalate (MEOHP), monobenzyl phthalate (MBzP), monobutyl phthalate (MBP), neonatal intensive care unit (NICU), phthalate, structural equation model

## Abstract

**Objective:**

We previously demonstrated that among 54 infants in neonatal intensive care units, exposure to polyvinyl chloride plastic medical devices containing the plasticizer di(2-ethylhexyl) phthalate (DEHP) is associated with urinary concentrations of mono(2-ethylhexyl) phthalate (MEHP), a DEHP metabolite. In this follow-up report, we studied the neonates’ exposure to DEHP-containing devices in relation to urinary concentrations of two other DEHP metabolites, and to urinary concentrations of metabolites of dibutyl phthalate (DBP) and benzylbutyl phthalate (BzBP), phthalates found in construction materials and personal care products.

**Measurements:**

*A priori*, we classified the intensiveness of these 54 infants’ exposure to DEHP-containing medical products. We measured three metabolites of DEHP in infants’ urine: MEHP and two of its oxidative metabolites, mono(2-ethyl-5-hydroxylhexyl) phthalate (MEHHP) and mono(2-ethyl-5-oxohexyl) phthalate (MEOHP). We also measured monobutyl phthalate (MBP), a metabolite of DBP, and monobenzyl phthalate (MBzP), a metabolite of BzBP.

**Results:**

Intensiveness of DEHP-containing product use was monotonically associated with all three DEHP metabolites. Urinary concentrations of MEHHP and MEOHP among infants in the high-DEHP-intensiveness group were 13–14 times the concentrations among infants in the low-intensiveness group (*p* ≤ 0.007). Concentrations of MBP were somewhat higher in the medium-and high-DEHP-intensiveness group; MBzP did not vary by product use group. Incorporating all phthalate data into a structural equation model confirmed the specific monotonic association between intensiveness of product use and biologic measures of DEHP.

**Conclusion:**

Inclusion of the oxidative metabolites MEHHP and MEOHP strengthened the association between intensiveness of product use and biologic indices of DEHP exposure over that observed with MEHP alone.

Phthalates are industrial chemical additives used primarily to soften and confer flexibility to plastics. In their pure form, phthalates are clear, oily liquids that are highly lipophilic and poorly soluble in water. They are a key component in a wide range of products, including but not limited to flexible polyvinyl chloride (PVC) plastics, vinyl tile, food packaging, insecticides, pharmaceuticals, and personal care products [Agency for Toxic Substances and Disease Registry (ATSDR) 2001, 2002; National Toxicology Program Center for the Evaluation of Risks to Human Reproduction (NTP-CERHR) 2003; Schettler 2006]. Because they are not chemically bound to the plastics, phthalates can be released as the products are used and discarded. The Centers for Disease Control and Prevention (CDC) measured urinary concentrations of phthalate metabolites in representative samples of the noninstitutionalized U.S. population, and for several phthalates, detectable levels of their metabolites were present in more than three-quarters of the participants (CDC 2005). These data confirm that the general population in the United States is exposed to phthalates.

Some animal studies suggest that at least some phthalates are developmental and reproductive toxicants (ATSDR 2001, 2002; NTP-CERHR 2000a, 2000b, 2003, 2005), although the effects on human health have not been extensively described, and the effective toxic doses of phthalates in these animal studies are many times higher than typical human exposures. Unusually high levels of exposure may occur, however, among infants admitted to neonatal intensive care units (NICUs), because of their small body mass and the use of phthalate-containing medical devices in their care (Calafat et al. 2004; Green et al. 2005). Of particular relevance is di(2-ethylhexyl) phthalate (DEHP), which is used as a plasticizer in a variety of medical products (U.S. Food and Drug Administration 2002), including those found in the NICU, such as bags containing blood, plasma, intravenous fluids, and total parenteral nutrition; tubing associated with their administration; nasogastric tubes; enteral feeding tubes; umbilical catheters; extracorporeal membrane oxygenation (ECMO) circuit tubing; hemodialysis tubing; respiratory masks and endotracheal tubes; and examination gloves. DEHP migrates out of the plastic into blood or other lipid-containing solutions in contact with the plastic, a phenomenon observed with blood stored in PVC bags (Peck and Albro 1982; Rock et al. 1984) and endotracheal tubes (Latini and Avery 1999). The rate of DEHP leaching depends on the type of solution in contact with the plastic material and on temperatures at the time of use, storage time, and percent DEHP in the plastic product (Marcel 1973). Overall, however, estimates of infants’ exposure to DEHP from common NICU devices exceed typical daily adult exposure [3–30 μg/kg body weight (bw)/day] (NTP-CERHR 2005) by one to two orders of magnitude, approaching the lowest observed adverse effect level (LOAEL) in animal studies (14–23 mg/kg bw/day) (NTP-CERHR 2005). The susceptibility of neonates in the NICU to high DEHP exposures may be compounded by their impaired ability to clear phthalates from their bodies. Until an age of 3 months, infants have immature glucuronidation pathways (Leeder and Kearns 1997). Because glucuronidation, via enzymes such as uridine diphosphate-glucuronosyltransferase, facilitates urinary excretion of phthalates and other xenobiotics, a reduced potential for glucuronidation may lead to slower excretion and higher concentrations of mono(2-ethylhexyl) phthalate (MEHP), a metabolite of DEHP, in neonates than in older children and adults. Neonates also have elevated gastric lipase activity (Hamosh 1990), which may enhance their ability to convert DEHP to MEHP.

We recently demonstrated that the intensiveness of DEHP-containing product use in NICU neonates is associated with higher exposure to DEHP, as reflected in elevated urinary concentrations of MEHP (Green et al. 2005). Although the median urinary concentration of MEHP among these neonates was five to seven times the median concentrations observed in the noninstitutionalized U.S. population (CDC 2005), 20% of these neonates had MEHP concentrations that were less than or equal to the limit of detection (LOD), 0.87 ng/mL. The absence of detectable levels of MEHP could have been caused by the relative absence of exposure to DEHP among these infants. However, MEHP is a minor urinary metabolite of DEHP. Oxidative metabolites, such as mono(2-ethyl-5-hydroxylhexyl) phthalate (MEHHP) and mono(2-ethyl-5-oxohexyl) phthalate (MEOHP), are more abundant in urine than is MEHP (Koch et al. 2004a, 2005). Specifically, MEHHP and MEOHP appear in urine at levels at least five times as great as does MEHP (Barr et al. 2003; Becker et al. 2004; CDC 2005; Kato et al. 2004; Koch et al. 2003b, 2004b; Silva et al. 2006) and thus may be more sensitive than MEHP as biomarkers of DEHP exposure.

In addition, as phthalate research has expanded, investigations have adopted a panel of several phthalate metabolites emanating from a range of parent phthalates (e.g., Hoppin et al. 2004; Swan et al. 2005). Although the metabolites can be analyzed separately, this approach does not acknowledge their biologic relationships and independencies. For example, exposure to DEHP results in elevations in urinary concentrations of MEHP, MEHHP, and MEOHP, but not monobutyl phthalate (MBP), a metabolite of dibutyl phthalate (DBP).

Therefore, in this follow-up report, we studied the intensiveness of the same 54 neonates’ exposure to DEHP-containing medical devices in relation to urinary concentrations of two oxidative metabolites of DEHP. We also evaluated urinary concentrations of monobenzyl phthalate (MBzP), a metabolite of benzylbutyl phthalate (BzBP), and MBP, a metabolite of DBP and a minor metabolite of BzBP (Anderson et al. 2001). Exposures to DBP and BzBP have not been evaluated for neonates in the NICU. Using a structural equation model (SEM), we integrated information from all five biomarkers to estimate the relations among use of DEHP-containing products, infants’ sex, and exposure to DEHP, DBP, and BzBP.

## Materials and Methods

### Study population

We studied a convenience sample of 54 infants enrolled from the level 3 NICUs at two major Boston, Massachusetts, area hospitals, as previously described (Green et al. 2005). Level 3 NICUs provide all newborn care, including mechanical ventilation, high-frequency ventilation, surgery, and cardiac catheterization. We selected infants to reflect a range of diagnoses (including congenital anomalies and developmental and metabolic abnormalities) and NICU care requirements (including ventilation, enteral feedings, parenteral nutrition, and indwelling catheterization). To be eligible for our study, infants must have been in the NICU at least 3 consecutive days before enrollment, have a corrected gestational age of ≤ 44 weeks, and have been born at or transferred to either hospital between 1 March and 30 April 2003. Concerns regarding the sensitivity of this research led to the design and implementation of a data and sample collection protocol that was based on visual inspection and did not include inspection of the medical records. The study protocol and methods were approved by the institutional review boards of Harvard School of Public Health, Brigham and Women’s Hospital, and Massachusetts General Hospital.

### Intensiveness of DEHP-containing product use

As previously described (Green et al. 2005), one of the study investigators took an inventory of products in use for the care of each infant. Before data collection, we defined three levels of intensiveness of DEHP-containing product use (low, medium, and high) based on a review of medical products typically used in both NICUs and information provided by their manufacturers with respect to DEHP content. Infants classified as having low-intensiveness product use were those receiving primarily bottle and/or gavage feedings. The medium-intensiveness group included infants receiving enteral feedings by indwelling gavage tubes either continuously or by bolus feedings; intravenous hyperalimentation by indwelling percutaneous intravenous central catheter (PICC) line, broviac, or umbilical vessel catheter (UVC); and/or nasal continuous positive airway pressure by nasal prongs. The infants in the high-intensiveness product use group included those receiving continuous indwelling umbilical vein catheterization, endotracheal intubation, intravenous hyperalimentation by the central venous route (i.e., PICC line, broviac, UVC), and an indwelling gavage tube (for gastric decompression). None of the infants changed product use groups over the course of observation.

### Assessment of phthalate metabolites in urine

The observing investigator collected spot urine samples at the end of each infant’s observation period(s). A total of 82 urine samples were collected from 54 infants. Two or more replicate samples were available for 18 infants, and of these, replicate samples were collected concurrently with the first samples from four infants (i.e., the diaper yielded enough urine for two vials), whereas 19 replicate samples were collected 6–72 hr after the first samples from 14 infants, the timing of which was determined by the investigator’s visiting schedule and the availability of urine in an infant’s diaper. We used these replicate samples to assess variability in urinary phthalate concentrations within individual infants.

Urine samples were collected by squeezing the urine either from a cotton gauze placed in the infant’s diaper at the beginning of the observation period or from the cotton filling of the diaper the infant wore during the period of observation. The cotton gauze or the removed cotton diaper filling was placed into a 3–5-cc polypropylene syringe (Becton-Dickinson, Franklin Lakes, NJ), the plunger was replaced, and the urine was squeezed into a 2- or 4-cc Nunc cryovial. Urine samples were frozen within 4–6 hr at −35°C and shipped on dry ice to the CDC for analysis.

Urine specimens were analyzed for 10 phthalate metabolites, including MEHP, MEHHP, MEOHP, MBP, and MBzP at the CDC (Atlanta, GA). Urinary concentrations of the five other metabolites were generally lower than the LOD for the amount of sample used in the analytical measurements, so we do not discuss these further. The analytical method, described in detail by Silva et al. (2004), involved the enzymatic deconjugation of the phthalate metabolites from their glucuronidated form, followed by automated solid-phase extraction. Reversed-phase high-performance liquid chromatography was used to separate the phthalate metabolites from other components in the extracted urine. The metabolites were quantified by isotope dilution–tandem mass spectrometry. Samples, reagent blanks, and quality control (QC) materials were processed identically. QC materials were analyzed along with the study samples to ensure the accuracy and reliability of the data. QC materials of low concentration (QCL) and high concentration (QCH) were prepared from a base urine pool, obtained from multiple anonymous donors, dispensed in 5-mL aliquots, and stored at −20°C. Each QC material was characterized by repeated measurements, spanned over several weeks, to define the mean concentrations and the 95% and 99% control limits of each phthalate metabolite. Each analytical run included QC materials, blanks, and unknown samples. The concentrations of the replicate QCH and QCL materials, averaged to obtain one measurement of QCH and QCL for each run, were evaluated using standard statistical probability rules. Specimens with urinary phthalate concentrations below the LOD were assigned a value of LOD divided by the square root of 2.

### Assessment of other variables

We collected data, as available, on gestational age and length of stay in the NICU. However, data on these variables were incomplete for about half of the infants because the sample collection was anonymous and not based on review of medical records. The interpretation of these data is tenuous because of missing data, so we do not present these results. The intensiveness of DEHP-containing product use correlates with the degree of support required and is in this way a rough surrogate measure for illness severity. Nonetheless, we were not able to distinguish levels of illness severity within group of product use, again because of the study’s anonymous design.

### Statistical analysis

To evaluate the changes in urinary phthalate concentrations in specimens collected from the same infant, we computed Spearman correlations between replicate urine specimens. For unadjusted comparisons of urinary phthalate concentrations by sex, institution, and product use group, we used the Kruskal-Wallis nonparametric test. In the first stage of analysis, we used multiple linear regression to compare urinary phthalate concentrations across product use groups, adjusting for institution and infants’ sex. To stabilize the variances of the urinary phthalates, we used log-transformed phthalate values as the dependent variables in the regression models. Because log-transformed values were used, the regression parameters in the model are interpreted as a proportion of urinary phthalate concentrations in the reference group for a given independent variable—for example, the urinary phthalate concentration of the medium-product-use group as a proportion of that in the low-product-use group. We fit one regression model for each of the five metabolites.

Although conventional multiple regression analyses produce informative results, several shortcomings motivated us to seek alternative methods. In particular, performing a separate regression analysis for each metabolite increases the probability of chance findings due to multiple testing, does not account for high correlations between phthalate concentrations (e.g., MEHHP and MEOHP), and yields a relatively large number of regression parameters to be interpreted. Further, because the sample size is small, it is of interest to use a statistical procedure that maximizes statistical power in testing the association between independent variables (product use, infants’ sex, and institution) and urinary phthalate metabolites. SEMs with latent variables (Sánchez et al. 2005) allow us to address these issues and, in addition, allow us to use replicate measurements of a given metabolite.

We fit an SEM with latent variables (Sánchez et al. 2005) as an alternative to the conventional regression analyses. This model assumes that there are two latent phthalate exposures that are indirectly measured by the five metabolites in the study, and that the latent variables are affected by (i.e., regressed on) the predictors of interest (product use, infants’ sex, and institution). Additional parameters (i.e., factor loadings) are estimated in order to translate between the outcomes’ scales. For example, a concentration of 1 ng/mL of MEHHP in a sample does not necessarily imply that the concentration of MEHP will also be 1 ng/mL.

**Figure 1:**
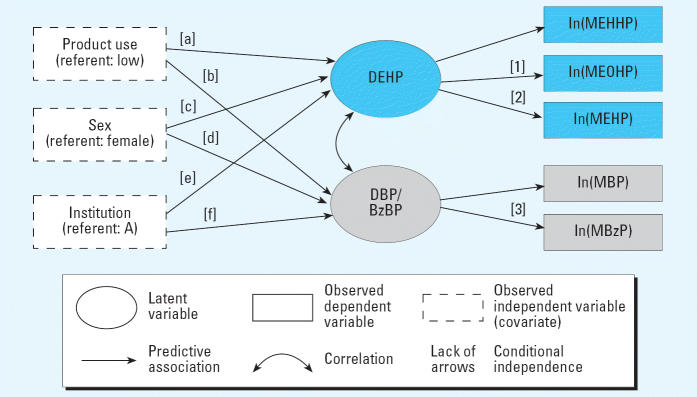
SEM describing associations we tested among external predictors and phthalate metabolites using two latent variables. The latent variable DEHP represents exposure to DEHP, which is measured by three urinary phthalate metabolites. The latent variable DBP/BzBP represents exposure to DBP and BzBP, which is measured by urinary MBP and MBzP. Corresponding analytic results are shown in Tables 5 and 6.

Figure 1 illustrates the model fitted, and the equations below specify the mathematical representation. The latent variable labeled “DEHP” can be said to represent the overall DEHP dose absorbed by the infant, where the underlying dose is measured indirectly by the metabolites MEHP, MEOHP, and MEHHP. Likewise, the latent variable labeled “DBP/BzBP” represents the overall dose of DBP and BzBP, which is measured indirectly by the metabolites MBP and MBzP. (Modeling a unique latent variable requires at least two measurements of that variable, so to represent DBP and BzBP as distinct entities in the SEM would have required additional measures of DBP and BzBP metabolites.) Formally, the model is:


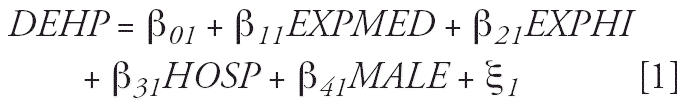



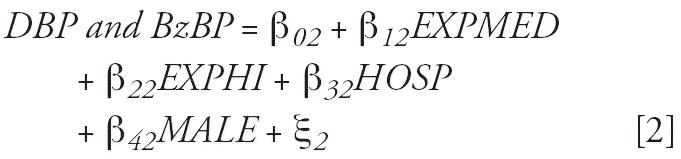



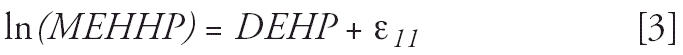



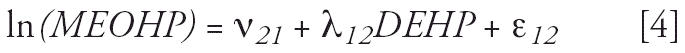



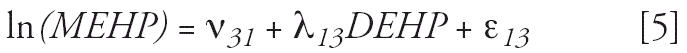



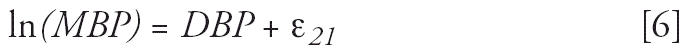



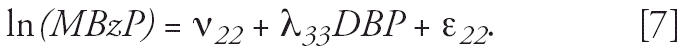


The β-values are the parameters of interest as they represent the effects of product use, sex, and institution on the latent phthalate exposures. Equations 3–7 imply that the metabolites measure the underlying parent phthalate exposures with error (ɛ values), and that the relations between the parent phthalates and metabolites can have different scales (λ values). For purposes of interpretation, the model assumes, in Equations 3 and 6, that *DEHP* is measured in ln*(MEHHP)* units and that *DBP* is measured in ln*(MBP)* units (e.g., Budtz-Jørgensen et al. 2002; Sánchez et al. 2005). Therefore, Equation 3 says that a one-unit change in *DEHP* results in one-ln*(MEHHP)*-unit change, whereas Equation 4 says one unit change in *DEHP* results in a λ*_12_*-unit change in ln*(MEOHP)*. Maximum-likelihood estimation for all model parameters was done in Mplus (Muthén and Muthén 1999–2004). Where available, we incorporated into our SEM analyses up to two replicate measurements per infant for a given metabolite.

## Results

As reported previously (Green et al. 2005), the 54 infants in our study were roughly equally distributed between the two institutions, and 34 (63%) were female. Thirteen (24%) infants were exposed to DEHP-containing products at low intensiveness, 24 (44%) were exposed at medium intensiveness, and 17 (32%) were exposed at high intensiveness. Among the first set of urine samples collected from these infants, only 9% had concentrations of MEHHP and MEOHP less than the LOD, compared with 20% who had concentrations of MEHP less than the LOD. Nearly all infants had detectable concentrations of MBzP. Maximum concentrations of MEHHP, MEOHP, MBP, and MBzP were 3,492, 3,376, 257, and 971 ng/mL, respectively. Overall, urinary concentrations of the three DEHP metabolites among the neonates in our study tended to exceed concentrations among persons ≥ 6 years of age, examined as part of the National Health and Nutrition Examination Survey (NHANES) (CDC 2005). Several studies, including NHANES, have reported somewhat elevated urinary concentrations of these metabolites among noninstitutionalized children in comparison with typical adult concentrations (Becker et al. 2004; CDC 2005; Koch et al. 2004b), but the median DEHP metabolite concentrations among the neonates in our study were even an order of magnitude higher than median concentrations found among these children (Table 1). The neonates’ urinary concentrations of MBP were slightly lower than those observed in other children and adult populations (Brock et al. 2002; CDC 2003), and although their concentrations of MBzP tended to exceed concentrations among the population overall, these concentrations were closer to those observed among other children (Brock et al. 2002; CDC 2005; Koch et al. 2005).

### Correlation between urinary phthalate metabolite concentrations in replicate urine samples

Among the infants with multiple urine samples, urinary phthalate measurements from the same infants were highly correlated. Spearman correlations between phthalate levels in the first and second specimens ranged from 0.61 (MBP; *p* = 0.007; *n* = 18) to 0.89 (MEHP; *p* < 0.0001; *n* = 17). Likewise, correlations between phthalate concentrations in the second and third specimens and first and third specimens tended to be strong as well (Table 2), although interpretation is limited by the small sample size (*n* = 5). We observed modest attenuation in the correlations between phthalate concentrations in urine specimens collected at different times compared with phthalate concentrations in specimens collected at the same time, although our data were limited to make a formal comparison (*n* = 4; data not shown).

### Correlations between different urinary phthalate metabolites

Urinary concentrations of the DEHP metabolites MEHP, MEHHP, and MEOHP were more highly correlated with each other than with the two other phthalate metabolites measured (Table 2). The correlation was particularly striking between MEHHP and MEOHP, two oxidation products of MEHP [Spearman correlation (*r*) = 0.95, *p* < 0.0001]. The DEHP metabolites were weakly correlated with MBP and MBzP, in contrast with the stronger correlation between MBP and MBzP (*r* = 0.80, *p* < 0.0001).

### Bivariate (unadjusted) associations between urinary phthalates and DEHP exposure group, sex, and institution

With increasing intensiveness of DEHP product use, we observed progressively higher concentrations of both MEHHP (*p* = 0.0002; Table 3) and MEOHP (*p* = 0.0003) in infants’ urine. These associations are consistent with the previously described findings for MEHP (*p* = 0.001). Median urinary MEHHP concentrations (and 25th and 75th percentiles) among infants in the low-, medium-, and high-intensiveness product use groups were 27 (18, 60), 307 (34, 614), and 555 (328, 844) ng/mL, respectively; median urinary MEOHP concentrations among infants in these groups were 29 (11, 42), 286 (25, 611), and 598 (318, 906) ng/mL, respectively. In contrast, in these unadjusted analyses, no consistent associations were present between intensiveness of product use and urinary concentrations of MBP and MBzP.

Similar to the pattern of associations between intensiveness of product use and urinary phthalates, urinary MEHHP and MEOHP (and MEHP) concentrations were significantly higher among infants at institution B (*p* ≤ 0.007), but concentrations of MBP and MBzP were similar across institutions. Overall, urinary phthalate concentrations did not vary substantially by infants’ sex.

### Adjusted associations between urinary phthalates and DEHP exposure group, sex, and institution

After adjusting for infants’ sex and institution of hospitalization, the association between intensiveness of DEHP-containing product use and both MEHHP and MEOHP persisted. Although the previously reported association between product use group and MEHP concentrations was strongly suggestive (*p* = 0.07), the associations between product use group and both MEHHP and MEOHP concentrations were highly significant (Table 4). Compared with infants exposed to DEHP-containing products at low intensiveness, infants exposed at medium intensiveness had urinary MEHHP concentrations that were 4.5 times as high [95% confidence interval (CI) of the multiplication factor, 1.2–16.5; *p* = 0.02], and infants exposed at high intensiveness had concentrations that were 14.1 times as high (95% CI of the multiplication factor, 3.3–61.0; *p* = 0.007). The association between product use group and urinary concentrations of MEOHP was similar in pattern and magnitude. We observed a suggestive pattern in the distribution of urinary MBP: Compared with infants exposed at low intensiveness, concentrations of MBP were nearly four times as high among infants exposed at medium (*p* = 0.05) and high intensiveness (*p* = 0.07). Concentrations of MBzP did not vary significantly by product use group. We obtained nearly identical findings when we conducted separate analyses for each institution.

The institutional pattern of DEHP metabolite distribution remained on adjustment as well. On average, urinary concentrations of MEHHP and MEOHP (and MEHP) among infants hospitalized at institution B were three times as great as concentrations among infants at institution A (*p*-value range, 0.03–0.06). Although male infants tended to have higher urinary concentrations of MEHP, this pattern was not apparent with respect to the two other DEHP metabolites or with respect to MBP and MBzP.

### Model of phthalate exposure incorporating all measured phthalate metabolites

The association between intensiveness of DEHP-containing product use and the latent variable representing DEHP exposure was monotonic and strongly significant (*p* = 0.001) (Figure 1, Tables 5 and 6). In units of MEHHP, the relative level of exposure to DEHP among infants in the medium-intensiveness product use group was 4.7 times that among infants in the low-intensiveness group (95% CI for multiplication factor, 1.5–15), and among infants in the high-intensiveness group, exposure to DEHP was 14 times as great (95% CI for multiplication factor, 3.9–50; Figure 1). The association between product use and DBP/BzBP did not follow a consistent pattern and was not significant (*p* = 0.2).

Infants at institution B had nearly three times the DEHP exposure (in units of urinary MEHHP) of infants at institution A (95% CI for multiplication factor, 1.1–7.3; *p* = 0.05). There were no significant differences in DEHP exposure by sex. The associations between DBP/BzBP and hospital or sex were not significant.

## Discussion

In our study of 54 infants receiving care in two NICUs, we found strong monotonic associations between the use of DEHP-containing medical devices and urinary concentrations of three metabolites of DEHP. These results reinforce and expand the association we reported previously with respect to MEHP (Green et al. 2005). Specifically, after adjustment for infants’ sex and institution, urinary MEHHP and MEOHP concentrations among infants who were exposed to DEHP-containing products at high intensiveness were > 13 times that of those in the low-intensiveness group. By comparison, as reported previously, concentrations of MEHP in the high-intensiveness group were five times that of those in the low-intensiveness group. Urinary MEHHP and MEOHP concentrations among infants who were exposed to DEHP-containing products at medium intensiveness were more than four times that of those in the low-intensiveness group. We established the specificity of the association between intensiveness of DEHP-containing product use and exposure to DEHP using an SEM, where we accounted not only for infants’ sex and institution, but also for urinary concentrations of two phthalate metabolites emanating from parent compounds other than DEHP, as well as up to two repeated measures of all of the urinary metabolites. The resulting parameters of this model confirmed the graded association between intensiveness of DEHP product use and direct evidence of exposure to DEHP, provided by the three DEHP metabolites. Comparably absent in these results was a monotonic association between DEHP product use and combined exposure to DBP and BzBP.

Exposure to DEHP was also higher among infants hospitalized at institution B than among infants hospitalized at institution A, independent from DEHP product use group. We speculate that this finding reflects the extensive use in particular of two DEHP-containing items at institution B: PVC indwelling endotracheal tubes, and a PVC indwelling hemodynamic monitoring UVC used for, among other things, parenteral nutrition. These DEHP-containing medical devices were sparingly used in institution A.

There has been interest in estimating the daily intake of DEHP using urinary concentrations of its metabolites and urinary creatinine. All 17 infants in the high-intensiveness product use group had urinary creatinine measurements concurrent with their urinary phthalate measurements. In turn, urinary creatinine measurements were available for only 62% and 92% of infants in the low- and medium-intensiveness product use group, respectively. Therefore, we estimated the DEHP dose for the infants in the high-intensiveness product use group. Using the formula expressed by Koch et al. (2003a) based on the method of David (2000), and assuming a uniform creatinine clearance rate of 9.8 mg/kg bw/day (Tietz 1990) and molar conversion factors (which relate urinary excretion of metabolite to diester ingested) of 0.23 (MEHHP) and 0.15 (MEOHP) (Koch et al. 2005), we estimated a median daily intake of DEHP among the infants in the high-intensiveness product use group ranging from 233 to 352 μg/kg bw/day based on MEHHP and MEOHP concentrations, respectively. This level of intake is one to two orders of magnitude above typical adult exposures and higher than the U.S. Environmental Protection Agency’s (EPA) reference dose of 20 μg/kg bw/day (U.S. EPA 2000), although still two orders of magnitude below the LOAEL observed in animal studies (14–23 mg/kg bw/day; NTP-CERHR 2005). The DEHP estimated dose for the infants in the medium-and low-intensiveness product use groups would be approximately 2- and 20-fold lower. Nonetheless, because of the assumptions used to compute ingested DEHP dose (e.g., uniform vs. individual-specific creatinine clearance rate and molar conversion factors calculated for one adult male after oral ingestion of DEHP), these estimates should be interpreted with caution.

In these first reported data on MBP and MBzP among neonates, we present two interesting findings. First, overall, although infants’ urinary concentrations of MBP were slightly lower than those observed in a recent survey of the U.S. population, their concentrations of MBzP were somewhat higher. For example, the median and 75th percentile concentrations of urinary MBzP in our infants were 41 and 131 ng/mL, versus 15.7 and 38.0 ng/mL in the U.S. population (CDC 2005). Moreover, the infants’ median MBzP concentration was nearly twice the median concentration observed among 36 toddlers, 3–7 years of age, in Germany (Koch et al. 2005). BzBP is commonly used in vinyl flooring, adhesives, sealants, car-care products, and some personal care products (NTP-CERHR 2003); and although some of these products, particularly vinyl flooring, may be present in the NICU, we did not specifically examine the sources and routes of these neonates’ exposure to BzBP for this study. Second, the modest association between medium- and high-intensiveness product use and urinary concentrations of MBP was unexpected. It is unlikely that the products we enumerated contained DBP; instead, the use of these products may have coincided with other exposures to DBP.

To date, few reports have described human neonatal exposures to phthalates (Calafat et al. 2004; Green et al. 2005; Hillman et al. 1975), and to our knowledge, no study has examined whether such exposures are associated with adverse health effects in these neonates. Limited data describe the effects of neonatal exposures to phthalates on health later in life. A study of 18 adolescents, 14–16 years of age, who had undergone ECMO as neonates found no apparent abnormalities in their growth and pubertal maturity, and the levels of luteinizing hormone, follicle-stimulating hormone, testosterone (in boys), and estradiol (in girls) were within the normal reference ranges for stage of pubertal development (Rais-Bahrami et al. 2004). It is difficult to draw definitive conclusions from this study, because it was very small, and normal reference ranges for reproductive hormones, physical growth, and sexual maturation are quite wide. Furthermore, the postnatal levels of DEHP or its metabolites at the time of the ECMO treatment were not known. Several recent epidemiologic studies suggest that phthalates may be human reproductive and developmental toxicants. In the only study, to our knowledge, that has examined phthalate exposure among infants (Main et al. 2006), 130 mothers in Denmark and Finland collected aliquots of their breast milk from infant feedings for several weeks starting 1 month after their sons were born. Concentrations of phthalate monoesters in the mothers’ breast milk were correlated with markers of Leydig cell function measured in the sons when they were 3 months old: Higher phthalate concentrations were correlated with higher sex-hormone binding globulin and ratio of luteinizing hormone to testosterone, and with lower free testosterone. Breast milk phthalate concentrations were not associated with cryptorchidism or markers of Sertoli cell function in the sons (Main et al. 2006). Other human reproductive and developmental toxicity studies have explored prenatal and adult exposures (Duty et al. 2003; Swan et al. 2005) and not early postnatal exposure.

Although data in humans are sparse as of yet, the toxicity of DEHP to testicular development and function has been well described in male animals (NTP-CERHR 2000a, 2005), and emerging data indicate that DEHP has hypoestrogenic effects in female animals (Lovekamp-Swan and Davis 2003); DEHP may impair the function of other organs, as well, including the kidney (ATSDR 2002). Although MEHP partially mediates DEHP’s reproductive toxicity (ATSDR 2002; Lovekamp-Swan and Davis 2003), preliminary evidence on the toxicity of MEHHP and MEOHP is mixed (Gray and Gangolli 1986; Stroheker et al. 2005). Appreciation of the potential effects of MEHP, MEHHP, and MEOHP is of particular relevance among infants because of their elevated gastric lipase activity (Hamosh 1990) (which promotes the digestion of milk fats and, concomitantly, the conversion of DEHP to MEHP) and impaired capacity for glucuronidation (Leeder and Kearns 1997) (which delays the excretion of DEHP and its metabolites). Because very low-birth-weight infants, resulting from preterm deliveries, comprise a large proportion of the NICU population, concern exists regarding risks from high perinatal DEHP exposure. Specifically, in the very preterm infant, DEHP may adversely affect male reproductive tract development, including testicular descent, which occurs late in the third trimester (Hutson et al. 1997).

Similar to DEHP and MEHP, DBP appears to exert adverse reproductive effects in male and female animals, mediated through its metabolite MBP (NTP-CERHR 2000b). Recent data suggest that neonatal exposure to DBP impairs the development of sex organs and alters the expression of steroid receptors in male rats (Kim et al. 2004). At very high doses, BzBP also is a reproductive toxin, particularly in male animals (NTP-CERHR 2003). In a study of 85 pregnant women (Swan et al. 2005), prenatal urinary concentrations of MBP and, to a lesser extent, MBzP were associated with significantly shorter anogenital distance in their infant sons. Inverse associations were also present for urinary MEHP, MEHHP, and MEOHP, but these were not statistically significant. This study examined prenatal exposures to phthalates, and still, as with DEHP, data on the health effects of human neonatal exposure to DBP and BzBP are limited.

The present study’s small size, narrow range of descriptive data on the infants, and lack of environmental measures from the infants’ NICU environment limited our ability to conduct more detailed analyses. Nonetheless, our study is the first to demonstrate that increasing intensiveness of DEHP-containing product use is directly proportional to biologic exposure to DEHP, reflected in stepwise elevations in urinary concentrations of three DEHP metabolites and in the absence of similar elevations in urinary concentrations of two phthalate metabolites from different parent compounds. It is also the first study to describe neonates’ exposure to DBP and BzBP.

In our SEM, we were unable to distinguish exposure to DBP from exposure to BzBP, limiting our ability to examine further the suggestive association observed in the conventional regression results between the two highest product use groups and urinary MBP. Such an examination would have required additional measures of DBP exposure, as was the case for DEHP. Yet the application of SEMs to our data revealed a potential drawback of using urinary MEHP as the sole biomarker of DEHP exposure. The measurement of urinary MEHHP and MEOHP concentrations added precision to estimated DEHP exposure, and given that these metabolites were detectable in more specimens, they may be more sensitive measurements of DEHP, as noted by others (Barr et al. 2003; Kato et al. 2004; Koch et al. 2003b, 2004a, 2004b, 2005; Silva et al. 2006). Nonetheless, given MEHP’s known biologic activity, it should continue to be measured in future studies.

## Conclusion

Measuring a panel of phthalate metabolites in the urine of neonates in two NICUs, we confirmed and demonstrated the specificity of the monotonic association between the intensiveness of DEHP-containing product use in their care and exposure to DEHP. High DEHP exposure levels in this sensitive population of patients and concerns about DEHP toxicity may justify routinely using available DEHP-free products that do not otherwise compromise the quality of care. We also reported a modest elevation in urinary MBP among infants receiving more intensive care. Future work should establish the sources and ramifications of these exposures, if any, on the development of similar neonates.

## Correction

In Table 3, the 75th percentile value for “Low” was incorrect in the manuscript published online; it has been corrected here.

## Figures and Tables

**Table 1:** Distribution of phthalate metabolites (in nanograms per milliliter unless otherwise specified).

		Phthalate parent compound/metabolite				
		DEHP			DBP	BzBP
	No.	MEHP	MEHHP	MEOHP	MBP^a^	MBzP
Distribution of first phthalate measurements (*n* = 54)						
LOD^b^		0.87	1.6	1.2	0.94	0.8
No. < LOD (%)		11 (20)	5 (9.3)	5 (9.3)	11 (20)	2 (3.7)
Geometric mean (SD)		15 (7.6)	133 (8.4)	120 (9.2)	14 (6.0)	43 (5.1)
25th percentile		3	32	28	7	14
Median		22	267	256	18	41
75th percentile		71	644	628	45	131
Median urinary phthalate concentrations from other studies						
United States
Children, 6–11 years of age^c^	393	4.4	32.9	22.6	40.0	37.0
Infants, 12–18 months of age (Brock et al. 2002)^d^	19	—e	NA	NA	29.0	20.2
Germany						
Children, 3–14 years of age (Becker et al. 2004)	254	7.2	52.1	39.9	NA	NA
Children, 3–7 years of age (Koch et al. 2004b, 2005)	36	6.6	49.6	33.8	NA	22.1
For comparison, persons ≥ 6 years of age^c^	2,782	4.1	20.1	14.0	26.0	15.7

NA, not available.

a MBP is also a metabolite of BzBP, albeit to a far lesser degree than of DBP.

b The LODs are the same across first, second, and third measurements.

c From NHANES, 2001–2002 (CDC 2005). Because this report provided separate estimates for mono-*n*-butyl phthalate and monoisobutyl phthalate, rather than a combined MBP value, we drew MBP estimates from the NHANES 1999–2000 report (*n* = 328 children and 2,541 total) (CDC 2003).

d Computed from reported results for each child.

e We did not compute a median for MEHP, because more than half of the children had levels < LOD, which was as high as 12 ng/mL.

**Table 2:** Correlations between repeated measurements of urinary phthalate metabolites and between different phthalate metabolites.

	Phthalate parent compound/metabolite				
	DEHP			DBP	BzBP
	MEHP	MEHHP	MEOHP	MBP^a^	MBzP
Spearman correlation between repeated measurements					
1st vs. 2nd (*n*)	0.89 (17)	0.85 (18)	0.80 (18)	0.61 (18)	0.81 (18)
2nd vs. 3rd (*n*)	0.90 (5)	0.90 (5)	1.00 (5)	0.87 (5)	0.90 (5)
1st vs. 3rd (*n*)	0.70 (5)	0.30 (5)	0.80 (5)	0.15 (5)	0.90 (5)
Spearman correlations between phthalate metabolites, among first measurements					
MEHHP	0.61				
MEOHP	0.56	0.95			
MBP	0.19	0.45	0.41		
MBzP	0.03	0.39	0.40	0.80	

a MBP is also a metabolite of BzBP, albeit to a far lesser degree than of DBP.

**Table 3:** Median (and 25th and 75th percentile) concentrations of urinary phthalate metabolites (ng/mL), by intensiveness of DEHP-containing product use, institution, and sex.

	Urinary MEHP				Urinary MEHHP				Urinary MEOHP				Urinary MBP				Urinary MBzP			
	25th	Median	75th	*p*-Value^a^	25th	Median	75th	*p*-Value^a^	25th	Median	75th	*p*-Value^a^	25th	Median	75th	*p*-Value^a^	25th	Median	75th	*p*-Value^a^
Intensiveness of DEHP-containing product use (*n*)																				
Low (13)	< LOD^b^	4	18		18	27	60		11	29	42		< LOD^b^	12	19		15	41	47	
Medium (24)	3	28	61		34	307	614		25	286	611		7	22	70		21	70	256	
High (17)	21	86	171		328	555	844		318	598	906		12	20	45		10	36	82	
				0.001				0.0002				0.0003				0.2				0.6
Institution (*n*)																				
A (28)	< LOD^b^	12	29		18	91	377		12	71	450		10	21	44		16	43	135	
B (26)	18	58	92		231	381	844		187	472	738		< LOD^b^	15	62		10	40	131	
				0.002				0.007				0.004				0.3				0.8
Sex (*n*)																				
Female (34)	3	20	64		32	267	644		36	286	598		10	18	35		28	50	131	
Male (20)	19	39	75		26	277	671		19	315	674		7	20	45		10	25	98	
				0.15				0.9				0.9				0.8				0.2
Overall	3	22	71		32	267	644		28	256	628		7	18	45		14	41	131	

25th and 75th are percentiles.

a From the Kruskal-Wallis nonparametric test for differences in phthalate distribution.

b LOD: 0.87 ng/mL for MEHP and 0.94 ng/mL for MBP.

**Table 4:** Adjusted relative concentration of urinary phthalate metabolite (95% CI), by intensiveness of DEHP-containing product use, sex, and institution.

	MEHP	MEHHP	MEOHP	MBP	MBzP
Intensiveness of DEHP-containing product use					
Low	1.0 (Referent)	1.0 (Referent)	1.0 (Referent)	1.0 (Referent)	1.0 ( Referent)
Medium	2.0 (0.6–6.9)	4.5 (1.2–16.5)	4.4 (1.1–17.3)	3.6 (1.0–13.0)	2.1 (0.6–6.9)
High	5.0 (1.3–20.0)	14.1 (3.3–61.0)	13.1 (2.8–61.3)	3.8 (0.9–16.2)	1.5 (0.4–5.9)
Overall differences among DEHP groups (*p*-value)	0.07	0.003	0.006	0.12	0.5
Infant’s sex					
Female	1.0 (Referent)	1.0 (Referent)	1.0 (Referent)	1.0 (Referent)	1.0 ( Referent)
Male	2.5 (0.9–6.8)	0.8 (0.3–2.3)	0.9 (0.3–2.6)	0.9 (0.3–2.7)	0.6 (0.2–1.5)
Institution					
A	1.0 (Referent)	1.0 (Referent)	1.0 (Referent)	1.0 (Referent)	1.0 ( Referent)
B	3.2 (1.1–9.0)	2.8 (1.0–8.5)	3.2 (1.0–10.0)	0.4 (0.1–1.1)	0.9 (0.3–2.5)

Estimates are multiplication factors derived from regression models of log-transformed urinary phthalates. They compare urinary levels of a given phthalate in the medium and high DEHP exposure groups with levels in the low DEHP exposure group, as well as comparing levels of a given phthalate by infants’ sex and institution. All comparisons are adjusted for the variables listed. See “Materials and Methods” for details.

**Table 5:** SEM-derived adjusted relative level of exposure to parent phthalates (95% CI), by intensiveness of DEHP-containing product use, sex, and institution.

	DEHP	DBP and BzBP combined
Intensiveness of DEHP-containing product use		
Low	1.0 (Referent)^a^	1.0 ( Referent)^d^
Medium	4.7 (1.5–15)^a^	2.5 (0.9–7.4)^d^
High	14 (3.9–50)^a^	1.8 (0.6–6.2)^d^
Infant’s sex		
Female	1.0 ( Referent)^b^	1.0 ( Referent)^e^
Male	0.8 (0.3–2.1)^b^	0.6 (0.3–1.4)^e^
Institution		
A	1.0 ( Referent)^c^	1.0 ( Referent)^f^
B	2.8 (1.1–7.3)^c^	0.8 (0.3–1.9)^f^

All comparisons were adjusted for the variables listed. The adjusted relative DEHP and DBP/BzBP exposures are expressed in the scales of urinary MEHHP and MBP concentrations, respectively. For example, after adjusting for sex and institution, exposure to DEHP among infants in the high product use group was about 14 times that among infants in the low product use group, using MEHHP concentrations as the scale. These results may be expressed in units of the other compounds by using the scales shown in Table 6; use of different scales does not alter the statistical significance of the results.

a Results correspond to arrow [a] in Figure 1.

b Results correspond to arrow [b] in Figure 1.

c Results correspond to arrow [c] in Figure 1.

d Results correspond to arrow [d] in Figure 1.

e Results correspond to arrow [e] in Figure 1.

f Results correspond to arrow [f] in Figure 1.

**Table 6:** Relative scale of phthalate metabolites sharing the same parent phthalate, derived from the SEM.

Metabolite	Relative scale
DEHP metabolites	
ln(MEHHP)	1
ln(MEOHP)	1.01^a^
ln(MEHP)	0.68^b^
DBP/BzBP metabolites	
ln(MBP)	1
ln(MBzP)	1.20^c^

In Table 5, the relative exposures to the parent compounds, DEHP and the combination of DBP and BzBP, are expressed in units of MEHHP and MBP, respectively. To express those results in the scale of a different metabolite, use the following: exp[(relative scale) × ln(adjusted relative parent compount exposure)], where relative scales are shown above.

a Results corresond to arrow [1] in Figure 1.

b Results corresond to arrow [2] in Figure 1.

c Results corresond to arrow [3] in Figure 1.
